# Evidence-based diagnosis and clinical practice guidelines for intestinal Behçet’s disease 2020 edited by Intractable Diseases, the Health and Labour Sciences Research Grants

**DOI:** 10.1007/s00535-020-01690-y

**Published:** 2020-05-07

**Authors:** Kenji Watanabe, Satoshi Tanida, Nagamu Inoue, Reiko Kunisaki, Kiyonori Kobayashi, Masakazu Nagahori, Katsuhiro Arai, Motoi Uchino, Kazutaka Koganei, Taku Kobayashi, Mitsuhiro Takeno, Fumiaki Ueno, Takayuki Matsumoto, Nobuhisa Mizuki, Yasuo Suzuki, Tadakazu Hisamatsu

**Affiliations:** 1 Department of Intestinal Inflammation Research, Hyogo College of Medicine, Hyogo, Japan; 2 Department of Gastroenterology and Metabolism, Nagoya City University Graduate School of Medical Sciences, Aichi, Japan; 3 Centers for Preventive Medicine, Keio University School of Medicine, Tokyo, Japan; 4 Inflammatory Bowel Disease Center, Yokohama City University Medical Center, Kanagawa, Japan; 5 Research and Development Center for New Medical Frontiers, Kitasato University, School of Medicine, Kanagawa, Japan; 6 Department of Gastroenterology and Hepatology, Tokyo Medical and Dental University, Tokyo, Japan; 7 Division of Gastroenterology, National Center for Child Health and Development, Tokyo, Japan; 8 Department of Inflammatory Bowel Disease, Division of Surgery, Hyogo College of Medicine, Hyogo, Japan; 9 Department of Inflammatory Bowel Disease, Yokohama Municipal Citizen’s Hospital, Kanagawa, Japan; 10 Center for Advanced IBD Research and Treatment, Kitasato University Kitasato Institute Hospital, Tokyo, Japan; 11 Department of Allergy and Rheumatology, Nippon Medical School Musashi Kosugi Hospital, Kanagawa, Japan; 12 Center for Gastroenterology and Inflammatory Bowel Disease, Ofuna Chuo Hospital, Kanazawa, Japan; 13 Division of Gastroenterology, Department of Medicine, Iwate Medical University, Iwate, Japan; 14 Department of Ophthalmology and Visual Science, Yokohama City University Graduate School of Medicine, Kanagawa, Japan; 15 Inflammatory Bowel Disease Center, Toho University Sakura Medical Center, Chiba, Japan; 16 Department of Gastroenterology and Hepatology, Kyorin University School of Medicine, Tokyo, Japan

**Keywords:** Intestinal behçet’s disease, Guideline, Evidence, Consensus, Behçet’s disease

## Abstract

Behçet's disease (BD) is an intractable systemic inflammatory disease characterized by four main symptoms: oral and genital ulcers and ocular and cutaneous involvement. The Japanese diagnostic criteria of BD classify intestinal BD as a specific disease type. Volcano-shaped ulcers in the ileocecum are a typical finding of intestinal BD, and punched-out ulcers can be observed in the intestine or esophagus. Tumor necrosis factor inhibitors were first approved for the treatment of intestinal BD in Japan and have been used as standard therapy. In 2007 and 2014, the Japan consensus statement for the diagnosis and management of intestinal BD was established. Recently, evidence-based JSBD (Japanese Society for BD) Clinical Practice Guidelines for BD (Japanese edition) were published, and the section on intestinal BD was planned to be published in English. Twenty-eight important clinical questions (CQs) for diagnosis (CQs 1–6), prognosis (CQ 7), monitoring and treatment goals (CQs 8–11), medical management and general statement (CQs 12–13), medical treatment (CQs 14–22), and surgical treatment (CQs 23–25) of BD and some specific situations (CQs 26–28) were selected as unified consensus by the members of committee. The statements and comments were made following a search of published scientific evidence. Subsequently, the levels of recommendation were evaluated based on clinical practice guidelines in the Medical Information Network Distribution Service. The degree of agreement was calculated using anonymous voting. We also determined algorithms for diagnostic and therapeutic approaches for intestinal BD. The present guidelines will facilitate decision making in clinical practice.

## Introduction

### The purpose and process of clinical practice guidelines for Behçet's disease

Behçet's disease (BD) is a systemic disease involving multiple organs. Therefore, the original Japanese edition of evidence-based clinical practice guidelines for systemic BD was developed by experts from several specialties, including extraprofessional fields, to optimize the understanding of systemic BD. The original JSBD (Japanese Society for Behçet's Disease) Clinical Practice Guidelines for BD (for systemic BD) were established in part by Health and Labour Sciences Research Grants for research on BD from the Ministry of Health, Labour and Welfare of Japan, and the section on intestinal BD was formulated as a collaborative project with the Research Group for Intractable Inflammatory Bowel Disease subsidized by the Ministry of Health, Labour and Welfare of Japan.

In 2007 and 2014, consensus statements for the diagnosis and management of intestinal BD were published by the Research Group for Intractable Inflammatory Bowel Disease subsidized by the Ministry of Health, Labour and Welfare of Japan [1, 2]. Subsequently, the present English edition of guidelines for intestinal BD, as a part of the original JSBD Clinical Practice Guidelines for systemic BD, were planned and translated for publication in an evidence-based style by using clinical questions (CQs).

### Treatment methods in guidelines for intestinal Behçet's disease

This evidence-based guidelines for intestinal BD complied with the clinical practice guidelines in the Medical Information Network Distribution Service (MINDS, 2007 version), which was financially supported by the Ministry of Health, Labour and Welfare of Japan. These guidelines were established to provide appropriate evidence for decision making by both patients and health-care providers.

Twenty-eight important CQs for clinical management were selected as unified consensus by the members of the committee. The statements and comments were formulated based on a search of published scientific evidence by these experts. However, limited data with a sufficient level of evidence, such as a randomized controlled trial or prospective cohort study, were available in this field. Accordingly, a systematic review was not performed, and the agreement of committee members was calculated to support the insufficient level of evidence. The levels of recommendation for each CQ were evaluated in accordance with the clinical practice guidelines in MINDS (Table 1).

**Table 1:** Classification of recommendation by the MINDS clinical practice guideline

	Level of recommendation	Contrast to evidence level	Degree of agreement
A	Strong recommendation to perform	Mainly 1	≥ 4.8
B	Recommendation to perform	Mainly 2, 3	≥ 4.5
C1	Consideration to perform with insufficient evidence	Mainly 4, 5, 6	≥ 4.0
C2	Inadvisability due to no evidence	No evidence	
D	Recommendation not to perform	Invalid or harmful evidence	

## Formal consensus- building

A round table discussion and the Delphi method for formal consensus building of these guidelines were performed because of the insufficient level of evidence for BD. Therefore, a consensus development conference was conducted to obtain agreement on the content of each CQ. Subsequently, the degree of agreement was calculated for each CQ using anonymous voting. Public comments to the draft were solicited from members of the Japanese Society of Gastroenterology and Research Group for Intractable Inflammatory Bowel Disease.

## Future issues

With the accumulation of new evidence and the approval of new therapeutic agents, including biologics, strategies for the management of BD will change considerably. The current guidelines will be updated triennially.

### Diagnosis


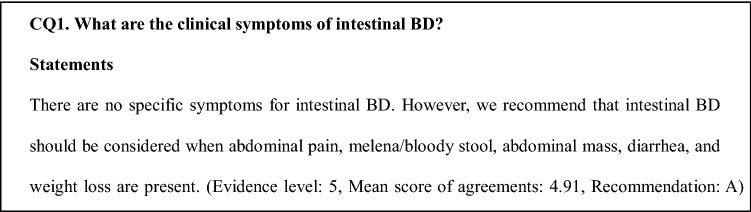


### Comments on CQ1

**Fig. 1:**
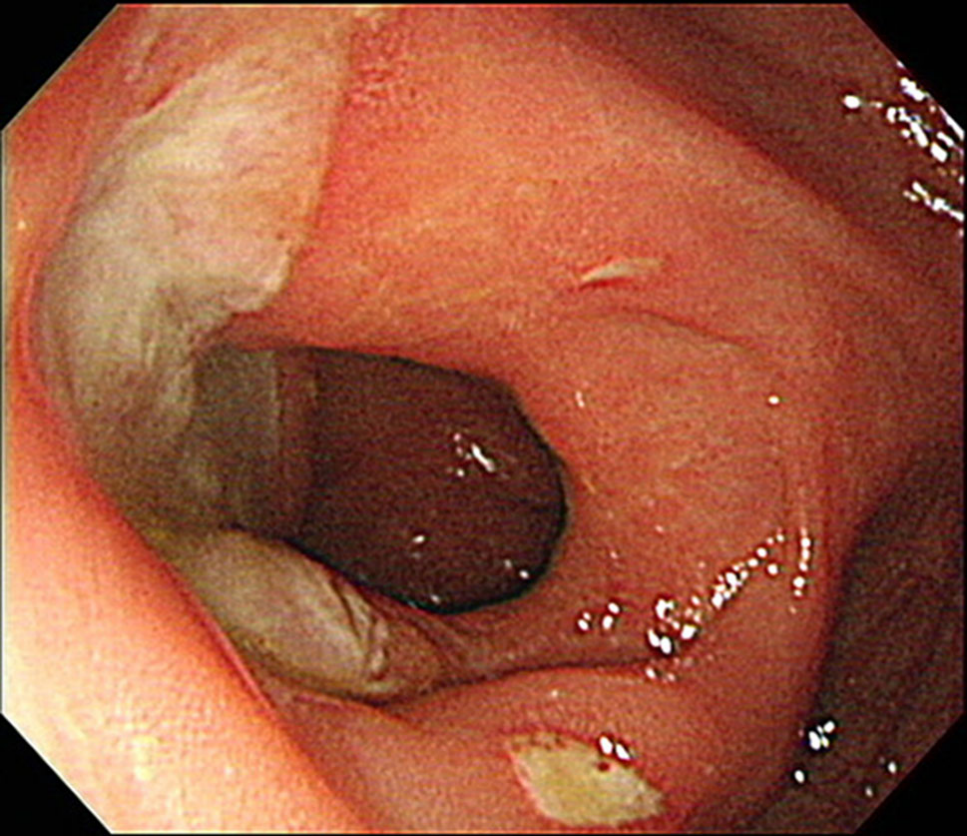
Typical ileocecal ulcer in a patient with Behçet’s disease

**Fig. 2:**
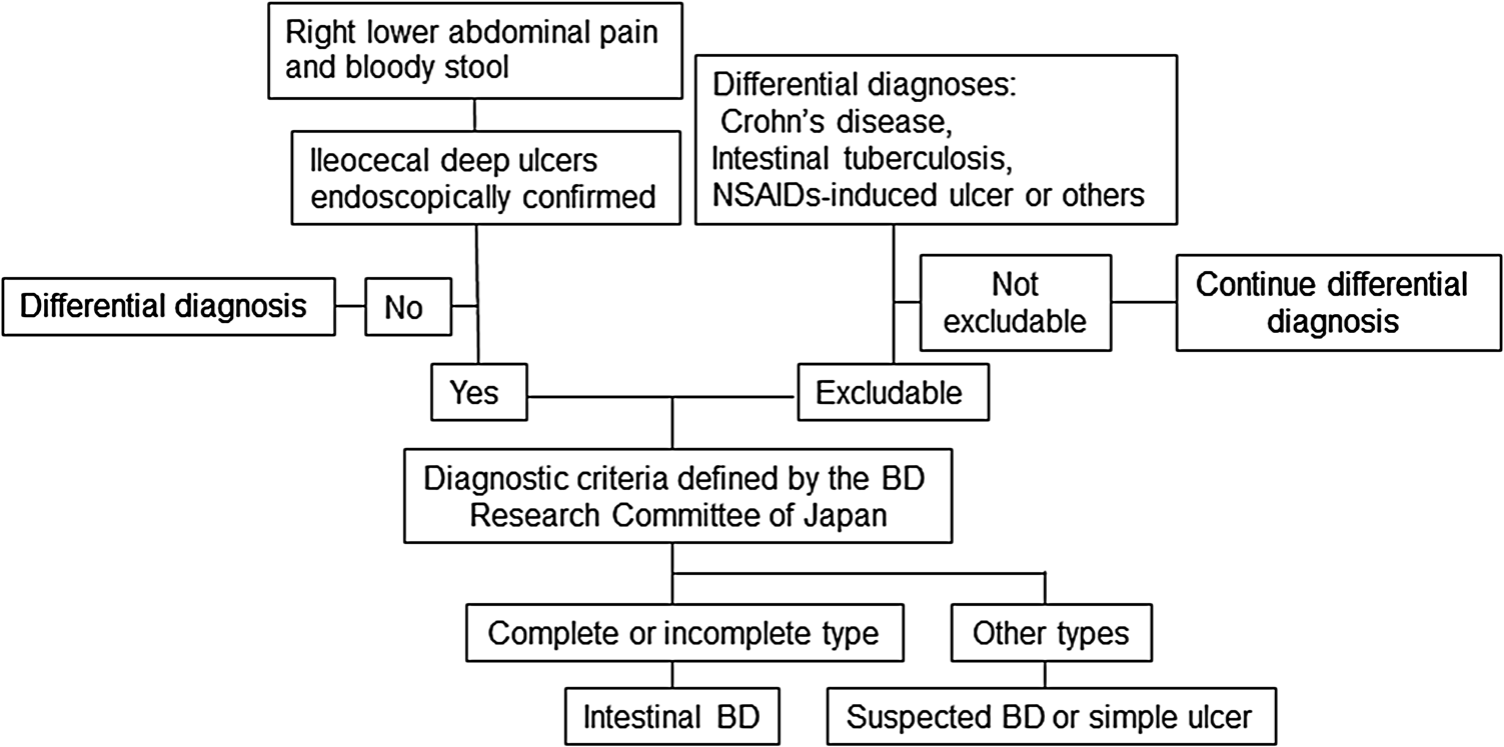
Algorithm for a definite diagnosis of intestinal BD. *BD* Behçet’s disease, *NSAIDs* non-steroidal anti-inflammatory drugs

Typically, volcano-shaped ulcers (Fig. 1) around the ileocecal region, right lower abdominal pain, and bloody stool are observed in intestinal BD (Fig. 2). Occasionally, patients experience severe abdominal symptoms as a result of ileus, perforation/penetration, and massive hemorrhage [2]. Intestinal BD is suspected when patients with BD (including suspected BD) present with these symptoms [2]. However, it is sometimes difficult to diagnose patients who have not been diagnosed with BD and do not present these symptoms. Differential diagnosis from other diseases, such as CD, is established while considering the presence/absence of local symptoms of BD, including recurring oral aphthae [2]. The diagnostic criteria for systemic BD (partial; Table 2a) and intestinal BD (Table 2b) are shown in Table 2 [3].

**Table 2:** Japanese diagnostic criteria for systemic BD (partial) and intestinal BD

a. Japanese diagnostic criteria for systemic BD (partial)
Main points
Main symptoms
Recurrent aphthous ulcers on oral mucosa
Skin lesions
a. Skin lesion with erythema nodosum
b. Subcutaneous thrombophlebitis
c. Follicular papules, acneform papules cf.) Skin hypersensitivity
Ocular lesions
a. Iridocyclitis
b. Posterior-uveitis (retinochoroiditis)
c. If the patients have the following eye symptoms after (a) and (b), diagnose as BD lesions in accordance with (a) and (b)
Posterior adhesion of iris, pigmentation on lens, retinochoroid atrophy, atrophy of optic nerve, complicated cataract, secondary glaucoma, leakage of bulbus oculi, genital ulcers Additional symptoms
Arthritis without deformity or sclerosis
Epididymitis
Gastrointestinal lesion represented by ileocecal ulceration
Vascular lesions
Central nervous system lesions moderate or severe
Criteria for diagnosis of disease types
Complete type
The four main symptoms appeared during the clinical course
Incomplete types
Three of the main four symptoms, or two main symptoms and two additional symptoms, appeared during the clinical course
Typical ocular lesion and another main symptom, or two additional symptoms appeared during the clinical course
BD suspected
Although some main symptoms appear, the case does not meet the criteria for the incomplete type
Typical additional symptom is recurrent or becomes more severe
Special lesions
Gastrointestinal lesions—presence of abdominal pain and occult blood should be confirmed
Vascular lesions—vasculitis of aorta, artery, large veins, or small veins should be differentially diagnosed
Neuronal lesions – presence of headache, paresis, lesions of brain and spinal cord, mental symptoms, and other symptoms should be confirmed
b. Japanese diagnostic criteria for intestinal BD
1. A volcano-shaped ulcer, circular or semicircular in shape, is typically observed in the ileocecal region under endoscopy or X-ray imaging. Conditions are met for the complete or incomplete type according to the diagnostic criteria for BD
2. Acute appendicitis and infectious enteritis are excluded based on clinical findings. Moreover, BD is differentiated from Crohn’s disease, enteric tuberculosis, and drug-induced enteritis based on clinical findings, endoscopic examination, and radiographic visualization

Patients who meet the above two criteria are diagnosed with intestinal BD. BD, Behçet’s disease

In a nationwide survey conducted in the fiscal year 2009, 12.3% of individuals had complete-type BD (excluding simple ulcers) and 15.1% showed ocular manifestations; these figures are relatively low within BD as a whole. Additionally, in a factor analysis of intestinal manifestations, the absence of ocular manifestations was significantly observed [4]. The proportion of patients with complete-type BD is decreasing within BD as a whole, but its association with intestinal manifestations remains unknown. Thus, complete-type BD and BD with ocular manifestations comprise a relatively small proportion of intestinal BD.
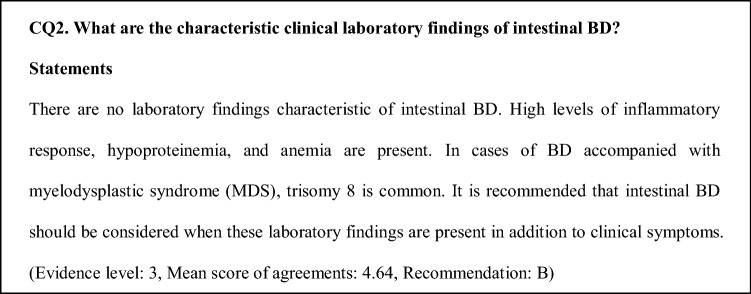


### Comments on CQ2

Blood biochemical tests do not show any characteristic findings, although high levels of inflammatory response including C-reactive protein (CRP), hypoalbuminemia, and anemia are observed, depending on the activity, nutritional status, and exhaustion level. A Korean group reported high CRP as a poor prognostic factor [5].

Genetic predisposition is considered an etiological factor of BD. With advancements in genome-wide association studies, single-nucleotide polymorphisms of *interleukin 23 receptor*, *interleukin 12 receptor subunit beta 2*, and *interleukin 10* have been reported as disease susceptibility genes, in addition to *human leukocyte antigen (HLA)-B51*. However, few studies on disease susceptibility genes associated with intestinal BD have been reported. The results of a meta-analysis showed that the *HLA-B51*-positive rate is lower in intestinal BD than in the other disease types [6]. For patients with intestinal BD, there are no detailed data on the prevalence of *HLA-A26*, which is included in the BD diagnostic criteria as a useful finding. However, patients with BD and accompanying MDS with trisomy have a high likelihood of presenting intestinal lesions [7, 8].
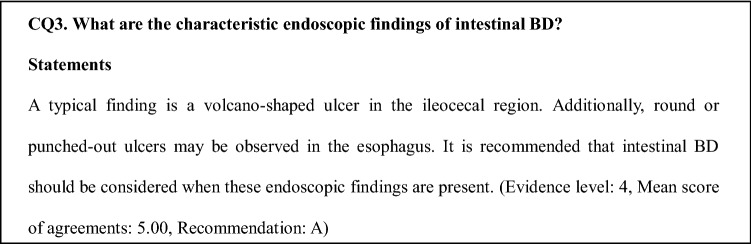


### Comments on CQ3

By definition, intestinal BD is diagnosed in patients that meet the diagnostic criteria proposed by the Ministry of Health, Labour and Welfare’s research team on BD and present with typical ulcerative lesions in the ileocecal region. Patients previously diagnosed with BD may also present with atypical lesions in areas other than the ileocecal region. These cases are not considered to represent intestinal BD.

Although not described in the international diagnostic criteria, there are several case–control and retrospective studies on gastrointestinal manifestations in patients with BD. Zou et al. performed upper and lower gastrointestinal endoscopy on 148 patients with BD and reported that intestinal manifestations were found in 35.1% of the patients, while active ulcers were found in the ileocecal region in 12.2% of the patients and in the esophagus in 2.7% of the patients [9]. When data from a small number of previous studies were consolidated, the incidence of gastrointestinal manifestations in patients with BD was found to be 12.2%–18.0% in the ileocecal region [10] and 2.7%–4.7% in the esophagus [11]. Conversely, 96% of patients with intestinal BD have been shown to have ulcerative lesions in the ileocecal regions [12].

Regarding the morphology and number of ulcerative lesions in patients with intestinal BD, 50.0%–83.3% are volcano-shaped ulcers, while round and geographic ulcers are also observed; 72.2%–76.0% are 1 cm or larger in diameter, and 50%–67% are single ulcers [9, 12, 13]. It has also been reported that, compared with CD, round ulcers, presence of five or fewer ulcers, intensive lesion distribution, and irregular geographic ulcers are findings suggestive of BD [14].

Esophageal lesions are common in the middle part of the esophagus. Single or multiple ulcers that are round or volcano shaped may lead to perforation and fistula formation. Differential diagnosis from lesions caused by cytomegalovirus and herpes virus is required [15].

Cheon et al. performed ileocolonoscopy in 145 patients with intestinal BD and reported lesions in the ascending colon in 12.4% patients, in the cecum in 10.3% patients, in the terminal ileum in 61.4% patients, and in the ileocecal valve in 42.1% patients [16]. Additionally, a capsule endoscopy study on intestinal manifestations showed that erosion and small ulcers were present in the distal small intestine. Further examination to determine the clinical significance of lesions in uncommon sites, including the upper gastrointestinal tract other than the esophagus, as well as how atypical cases should be treated.

Occasionally, “chronic punched-out ulcers around the ileocecal region” and “Ul–IV ulcers round or oval in shape with clearly defined borders that show a strong undermining tendency”, without BD’s symptom, are termed “simple ulcers” [17, 18]. This is a disease concept unique to Japan. Differences/similarities between simple ulcers and intestinal BD, treatment response according to the presence/absence of accompanying oral aphthae, and long-term disease course are being investigated. However, there is confusion regarding these issues, including their definitions.
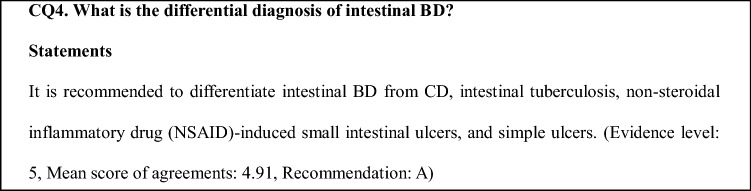


### Comments on CQ4

In endoscopic imaging, intestinal BD typically appears as an oval punched-out ulcer with well-defined margins in the ileocecal region. Deep ulcerative lesions are often referred to as “volcano-shaped ulcers.” Diseases in which ulcerative lesions manifest in the ileocecal region include CD and intestinal tuberculosis. CD exhibits characteristic endoscopic images that show longitudinal ulcerations and a cobblestone appearance, and is differentiated from intestinal BD based on the presence of skip lesions and anal lesions. However, in the absence of these typical lesions, it may be difficult to differentiate between intestinal BD and CD based on endoscopic findings alone; hence, systemic symptoms and other clinical/laboratory findings must also be taken into account [19].

Differentiation from intestinal tuberculosis is important in determining treatment policy. In particular, active intestinal tuberculosis must be eliminated when using tumor necrosis factor (TNF) inhibitors. Typical endoscopic findings of intestinal tuberculosis include ring-shaped ulcers and scarred areas with discoloration. However, endoscopic images of intestinal tuberculosis can vary in presentation, and care is therefore required during diagnosis. Medical interviews and findings on physical examination, chest X-ray, or chest computed tomography (CT); interferon-gamma release testing (QuantiFERON, T-SPOT), and Mantoux testing are used for the differential diagnosis of intestinal tuberculosis.

Simple ulcers are a disease concept proposed in 1979 by Muto as “chronic punched-out ulcers around the ileocecal region” and by Watanabe et al. as “Ul–IV ulcers round or oval in shape with well-defined margins that show a strong undermining tendency, appear frequently on or near the ileocecal valve, and, from a histological perspective, are chronically active and show signs of nonspecific inflammation.” It is difficult to differentiate intestinal BD from simple ulcers based on endoscopic and pathological findings alone. Currently, the presence/absence of clinical features of systemic BD is what distinguishes simple ulcers from intestinal BD. Based on diagnostic criteria proposed by the Ministry of Health, Labour and Welfare’s research team, intestinal BD is defined as cases meeting the criteria for complete- and incomplete-type BD. Therefore, patients presenting with typical oval volcano-shaped ulcerative lesions in the ileocecal region and oral aphthae alone cannot be diagnosed as having intestinal BD, but should instead be diagnosed as having simple ulcers or suspicion of intestinal BD. However, clinical features of BD may appear after a time lapse, and a patient may meet the diagnostic criteria for intestinal BD during follow-up.

NSAID-induced small intestinal ulcers rarely present as volcano-shaped ulcerative lesions in the ileocecal region. However, multiple ulcerative lesions induced by NSAID therapy may occasionally require differential diagnosis from intestinal BD [2]. History of internal use of NSAIDs and amelioration of ulcerative lesions as a result of discontinuing NSAIDs are the most important points when establishing a differential diagnosis.

Nonspecific multiple small intestinal ulcers, a condition that causes multiple ulcerative lesions in the ileum, may also require differentiation from intestinal BD. This condition develops relatively early in life, is more common in women, and has clinical characteristics that include chronic iron deficiency anemia and hypoalbuminemia. It has been shown to be an autosomal recessive disease caused by a mutation in the *SLCO2A1* gene, which codes for a prostaglandin transporting protein [20].
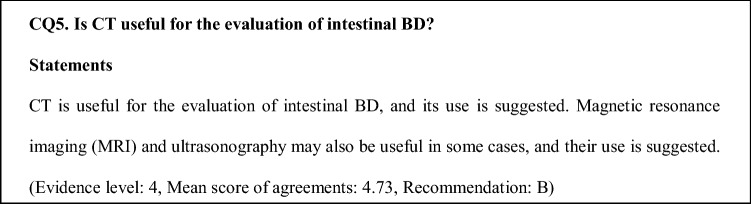


### Comments on CQ5

Contrast-enhanced CT is beneficial in evaluating the disease state as intestinal BD may exhibit intestinal wall thickening, inflammatory masses, penetration, and perforation. Contrast-enhanced CT may be used as the first choice for patients suspected to have abscess formation or perforation with severe right lower abdominal pain or inflammatory masses. MRI is also useful for the visualization of intestinal wall thickening and inflammatory masses. Furthermore, the effectiveness of CT enterography and MR enterography for differentiating intestinal BD from intestinal tuberculosis and CD has been reported [21]. Although abdominal ultrasonography is influenced by the skill level of the operator and presence of gastrointestinal gas, it can visualize intestinal wall thickening and inflammatory masses and is minimally invasive. However, cross-sectional imaging of CT, MRI, and abdominal ultrasonography is not suitable for the morphological diagnosis of ulcerative lesions, meaning that gastrointestinal angiography and endoscopy or findings based on surgical specimens are required for a definitive diagnosis.

Frequent CT scans may put patients with intestinal BD at risk of radiation exposure [22]. Unnecessary tests should therefore be avoided, while use of other modalities (MRI, abdominal ultrasonography) should be considered.
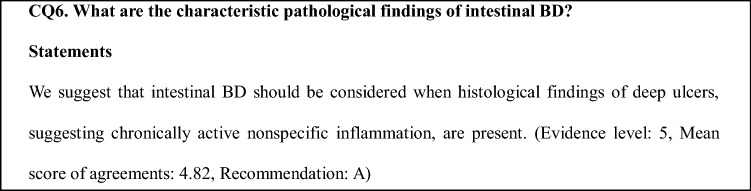


### Comments on CQ6

Histological findings show deep ulcers indicative of the presence of chronically active nonspecific inflammation. The ulcer floor consists of three layers, as follows: chronic diffuse inflammatory cell infiltration primarily composed of neutrophils, a necrotic layer, lymphocytes, and plasma cells; a granulation tissue layer rich in capillaries; and a fiber tissue layer containing a small number of chronic inflammatory cells and copious fibroblasts [2]. A typical lesion has a flat base that is wider than other areas, giving it a flask-like shape. In the ulcer margin, chronic active inflammatory cell infiltration in the mucosa is found in small areas around the ulcer, and it is accompanied by neogenesis of the capillaries, a decrease in the number of glandular ducts, disordered arrangements, and epithelial cell rejuvenation. Intestinal BD differs from CD in that aggregated lymphocytes are confined to the ulcer floor and its vicinity, and inflammatory cell infiltration in the mucosa around the ulcer is minor. Since there are no specific mucosal findings, it is difficult to actively diagnose intestinal BD based on endoscopic biopsy.

### Prognosis


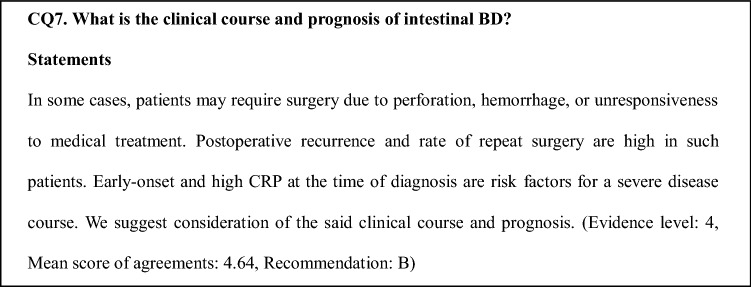


### Comments on CQ7

In several cases, patients with intestinal BD require emergency surgery due to perforation and hemorrhage and have high postoperative recurrence and repeat surgery rates. Therefore, some researchers consider intestinal lesions in BD to be a poor prognostic factor. In a study that examined the disease course of 130 patients with intestinal BD over the course of 5 years after diagnosis, disease activity patterns where remission or mild disease activity was maintained showed the highest percentage of 56.2%. However, 16.2% of patients experienced multiple relapses or persistent subjective symptoms. In a group of patients with a severe clinical course, factors such as young age at the time of diagnosis, high erythrocyte sedimentation rates and CRP, high disease activity (Disease Activity Index for Intestinal BD [DAIBD]), and hypoalbuminemia were extracted [23]. Additionally, in a study in 291 patients, those diagnosed at a young age were found to have followed a severe disease course and, in terms of gender difference, although some clinical symptoms tended to be severe in male patients at diagnosis, there was no clear gender difference in the disease course [24]. Moreover, it has been reported that being under the age of 35 years, high CRP (≥ 1.5 mg/dl), and high disease activity (DAIBD ≥ 60) are risk factors for resistance to 5-aminosalicylic acid (ASA)/sulfasalazine [5]. Naganuma et al. reported that ileac lesions and accompanying ocular manifestations are risk factors for surgery and that an increased CD8 ^+^ DR ^+^ lymphocyte ratio poses a risk of recurrence [25].

Kimura et al. analyzed 34 patients with intestinal BD and reported *HLA-B51* positivity, high CRP levels, high white blood cell count, and bloody stool as refractory factors that led to the administration of corticosteroids or treatment involving drugs, including 5-ASA or more aggressive treatments (immunomodulatory drugs and TNF inhibitors) [26].

Regarding surgery, in a study involving 136 Japanese patients, male patients were found to require surgery more frequently, with more undergoing emergency surgery due to perforation compared with female patients [27]. Postoperative recurrence can occur at a relatively early stage, often within 2 years [25–27], and the cumulatively recurrence rate is as high as 30%–75% over 2 years [24, 28].

### Monitoring and treatment goals


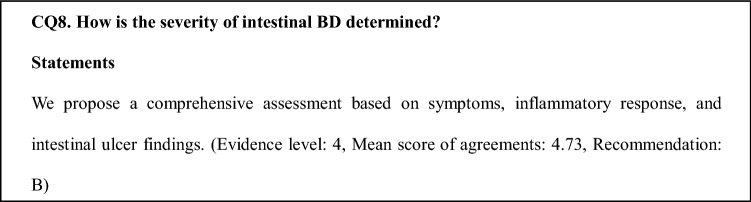


### Comments on CQ8

Currently, criteria for determining the severity of intestinal BD have not been established. It is required that actual severity is determined comprehensively based on the presence/absence of systemic symptoms, such as fever and extraintestinal manifestations, abdominal findings (degree of abdominal pain, presence/absence of inflammatory masses and rebound tenderness), depth of ulceration, presence/absence of intestinal complications (hemorrhage, stenosis, fistulas), inflammatory response (CRP, white blood cell count, erythrocyte sedimentation rate), and degree of anemia [2]. Additionally, the new DAIBD, proposed by a Korean group, has been reported as effective in determining disease severity. However, DAIBD has not yet been fully validated. In DAIBD, general status, fever (38 °C or higher), extra-intestinal findings, degree of abdominal pain within 1 week, presence of abdominal mass, degree of tenderness, intestinal complications (fistulas, perforation, abscesses), and the number of times a patient passed watery stool within 1 week are assessed. Remission is defined as a score lower than 19, mild disease as ranging between 20 and 39, moderate as ranging between 40 and 79, and severe as a score equal to or higher than 75 [29]. Endoscopic severity has a weak association with DAIBD (*r* = 0.434), but the number of intestinal ulcers and volcano-shaped ulcers described as crater-like intestinal ulcers is a potential predictor of severity on DAIBD [30, 31].
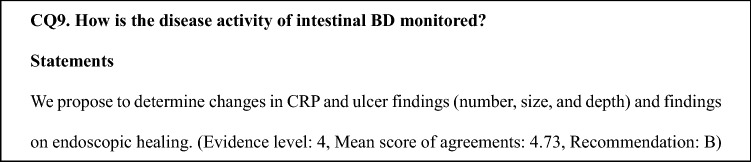


### Comments on CQ9

Inflammatory response (CRP) is one of the biomarkers used to evaluate the degree of intestinal inflammation and the activity of BD [32]. Furthermore, changes in ulcerative findings under endoscopy and findings on endoscopic healing allow direct evaluation of the improvement in disease activity of intestinal lesions. Additionally, the use of a disease activity index (DAIBD) as a factor for monitoring disease activity has been proposed. In this report, DAIBD has been shown to be effective for monitoring changes over time by evaluating changes in score as improved (≥ 20), somewhat improved (10–19), no change (− 9 to 10), somewhat deteriorated (− 14 to − 11), and deteriorated (≤ − 15) [29]. However, DAIBD has not yet been fully validated. However, basic research has shown that the expression of soluble triggering receptor on myeloid cell-1 was associated with DAIBD (*r* = 0.762) and CRP (*r* = 0.383) [32].
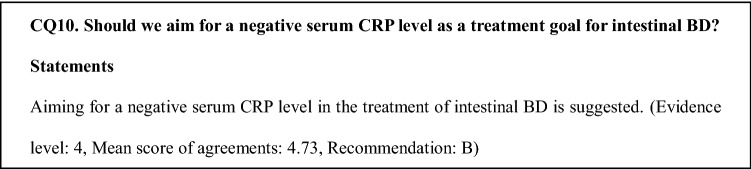


### Comments on CQ10

Currently, there are no prospective studies demonstrating that aiming for a negative CRP level improves the prognosis (recurrence rate, surgical rate) of intestinal BD. However, it has been reported in a retrospective study that the recurrence rate was high in patients whose CRP levels were elevated (≥ 4.4 mg/dL) after surgery compared with those whose CRP levels were low (< 4.4 mg/dL) [28]. In a clinical trial of adalimumab in Japan, it was reported that serum CRP levels were lower in patients who showed endoscopic healing [33]. In a clinical trial of infliximab targeting Japanese patients with special-type BD, CRP decreased as clinical symptoms improved [34]. CRP was also used as an efficacy index in a clinical study that compared the efficacy of prednisolone and etanercept [35]. In a consensus statement by experts, negative serum CRP is considered a possible treatment goal in clinical practice [2]. However, there are no verification studies that report the positive effects of negative CRP, such as improvement in prognosis. In some cases, active ulcerative lesions continue to be observed under endoscopy, even after the patient has tested negative for CRP.
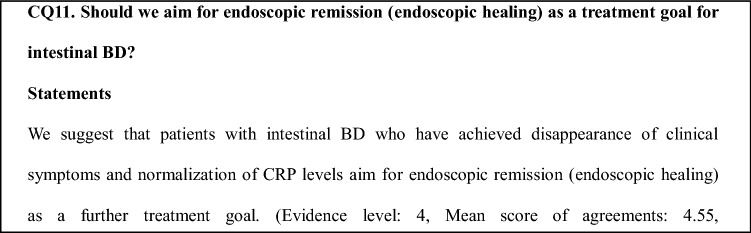


### Comments on CQ11

Although the definition of remission in intestinal BD is yet to be established, disappearance of clinical symptoms and normalization of CRP levels are the first steps in the treatment. Choi et al. followed up on 43 patients with intestinal BD for an average of 73 months and reported that complete remission was induced in 38% of patients at 8 weeks after the start of treatment, that these patients showed significantly lower surgery rates (*p* = 0.028) compared with those in which remission was not induced, and that gastrointestinal lesion relapse rates in patients where complete remission was induced were 25% and 49% at 2 and 5 years, respectively [36]. There are also studies that report high CRP levels as a poor prognostic factor [28, 37]. However, active lesions continue to be observed under endoscopy in many cases, even in patient in clinical remission. Lee et al. reported a weak association (*r* = 0.434) between endoscopic activity and DAIBD [30], while Yim et al. reported, in a study in 80 patients with intestinal BD, that endoscopically active ulcers were observed in 57% patients in clinical remission [38].

In recent years, the number of endoscopic studies on the effects of TNF inhibitors has increased [39, 40]. In a study conducted in Japan that examined 20 patients with intestinal BD who were administered adalimumab, marked clinical improvement rates of 45% and 60% were observed at 24 and 52 weeks, respectively, while endoscopic mucosal healing rates were 45% and 55% at 24 and 52 weeks, respectively [33]. Furthermore, in a study conducted in Japan in which infliximab and methotrexate were administered in combination to ten patients with intestinal BD resistant to existing treatments, it was reported that 50% and 90% of ileocecal ulcerative lesions disappeared at 6 and 12 months, respectively [41].

Patients in endoscopic remission (endoscopic healing) are expected to have a better prognosis than patients in clinical remission with residual endoscopically active lesions. However, the necessity of endoscopic healing in view of each patient’s clinical course and the risk, in terms of adverse events due to enhanced treatment must be comprehensively considered to determine whether endoscopic mucosal healing as a higher treatment goal should be achieved after the induction of clinical remission.

### Medical management and general statement


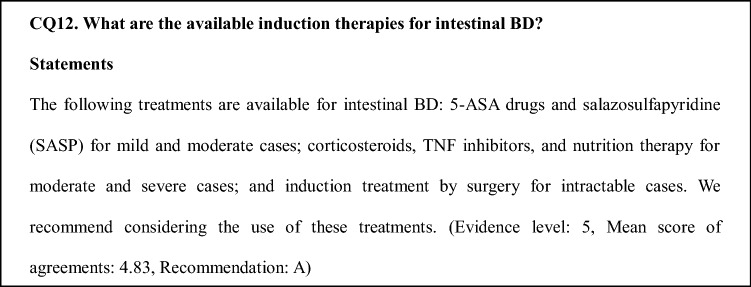


### Comments on CQ12

**Fig. 3:**
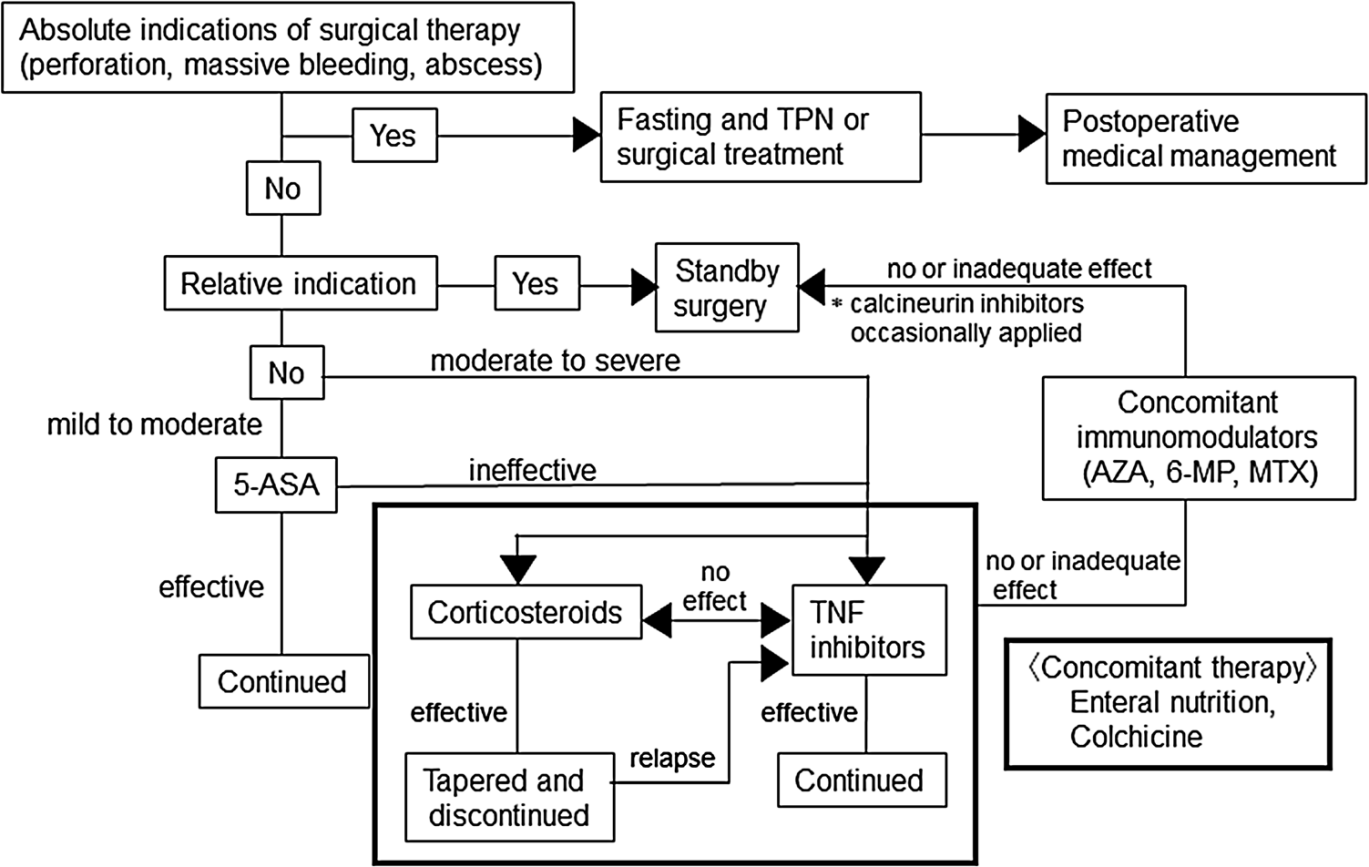
Algorithm for the treatment of intestinal BD. *5-ASA* 5-aminosalicylic acid, *6-MP* 6-mercaptopurine, *AZA* azathioprine, *MTX* methotrexate, *TNF* tumor necrosis factor, *TPN* total parenteral nutrition

5-ASA drugs and SASP may be effective for mild and moderate cases with gastrointestinal symptoms such as abdominal pain, diarrhea and melena, and mild systemic symptoms (Fig. 3) [5, 42].

Consider the administration of corticosteroids as induction treatment if the activity level of intestinal lesions is moderate or higher, if the induction of remission by other treatments is insufficient, or if the patient experiences severe systemic symptoms [43, 44].

Consider the administration of TNF inhibitors if corticosteroids are not effective or as a treatment to prevent the administration of corticosteroids [33, 34, 40, 45, 46].

Occasionally, colchicine is administered empirically, but there is insufficient evidence regarding its administration.

Enteral nutrition therapy with elemental diets may be effective in inducing remission, and is indicated for patients resistant to drug therapy and those with serious intestinal disorders such as stenosis.

Although scarce, there are reports on the efficacy of calcineurin inhibitors (oral tacrolimus) [47].

Consider induction treatment by surgery for intractable cases resistant to medical treatment and cases of fistula formation.
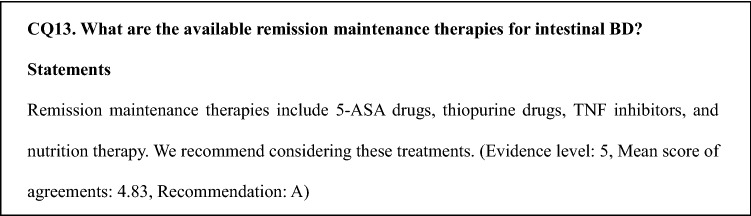


### Comments on CQ13

5-ASA drugs may be used as maintenance therapy for intestinal lesions if symptoms are relieved following induction therapy [4].

Consider the administration of immunomodulators (thiopurine/methotrexate) as remission-maintenance therapy in patients resistant to treatment with corticosteroids and TNF inhibitors and if symptoms recur during the gradual reduction of corticosteroids [42, 48, 49].

Occasionally, colchicine is administered empirically, but there is insufficient evidence regarding its administration.

Transition to enteral nutrition may be considered for patients whose symptoms have improved following total parenteral nutrition or fasting.

Patients who respond to TNF inhibitors will subsequently be transitioned to maintenance administration [33, 41, 50, 51].

### Treatment (medical treatment)/detailed discussion


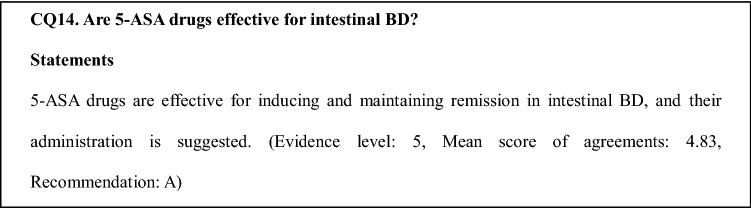


### Comments on CQ14

No studies have provided sufficient evidence on the efficacy of 5-ASA drugs for inducing or maintaining remission in intestinal BD. In a case series conducted by a Korea study group, 5-ASA drugs were reported to be effective for induction of clinical remission and maintenance of remission [5]. In Japan, only case reports of effective cases are available. The “Draft revision for the clinical consensus statement on intestinal BD” [2] was prepared in 2012 by a research team led by Prof. Hibi to understand the actual state of small intestinal ulcers of unknown cause and to establish disease concepts, epidemiology, and a treatment system as part of specific disease countermeasure research conducted with grants-in-aid for scientific research provided by the Ministry of Health, Labour and Welfare and partially revised in 2013. In this statement, 5-ASA drugs are considered standard treatment for intestinal BD. It is also stated that 5-ASA drugs may be effective for induction therapy in mild to moderate active cases and that 5-ASA drugs and colchicine may be used as maintenance therapy for patients who have undergone clinical remission. The optimum dose of 5-ASA drugs is 2.25–3 g/day and 3–4 g/day for cs0012and SASP, respectively. Statements on 5-ASA drugs are prepared based on the consensus among specialists. Although 5-ASA drugs are relatively safe and are suitable for maintenance therapy, the frequency and characteristics of effective cases and the treatment effects on intestinal lesions are unknown.
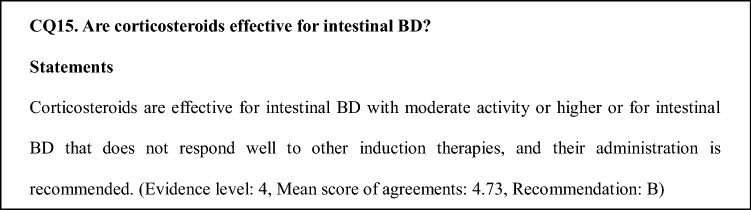


### Comments on CQ15

This recommendation is based on experience accumulated in actual clinical practice. However, data showing high levels of evidence for the efficacy rate of corticosteroids are limited in the literature. Additionally, adverse events caused by long-term administration of corticosteroids and their combined use with other immunosuppressive treatments require caution.

Corticosteroids are used as part of induction therapy for intestinal BD with moderate or higher activity and when induction therapy by other treatments is not sufficiently effective [31, 37, 52]. In the Japanese consensus, it is stated that 0.5–1.0 mg/kg of prednisolone should be administered for 1–2 weeks and that its dose should be reduced by 5 mg each week [1, 2]. Moreover, in severe cases that require hospitalization, corticosteroid pulse therapy, which involves intravenous administration of prednisolone and administration of 1 g of methylprednisolone for 3 days, is considered [53, 54]. Consistent with the start of administration of TNF inhibitors, screening tests, including those for tuberculosis and hepatitis B, are required prior to the administration of high doses of corticosteroids. It has been reported in a retrospective study that the efficacy rates of corticosteroids in inducing remission are 46%, 42%, and 11% for remission, effectiveness, and ineffectiveness, respectively [43]. However, there are no data from prospective studies involving a large number of cases. The retrospective study reported that, after 1 year, 35.2% of the patients in whom remission was induced were addicted to corticosteroids, and 7.4% underwent surgery [43].

The administration of TNF inhibitors for corticosteroid-resistant cases and immunomodulators for cases of corticosteroid addiction should be considered for intractable cases. Adverse effects involving the eyes, bones, and adrenal glands following long-term administration of corticosteroids must be avoided. However, a relatively large number of patients experience a relapse during corticosteroid tapering, and dose reduction can only be performed slowly in some patients. Additionally, combined administration of co-trimoxazole is considered, since high doses of corticosteroids and their combined use with other immunomodulators increase the risk of opportunistic infection.
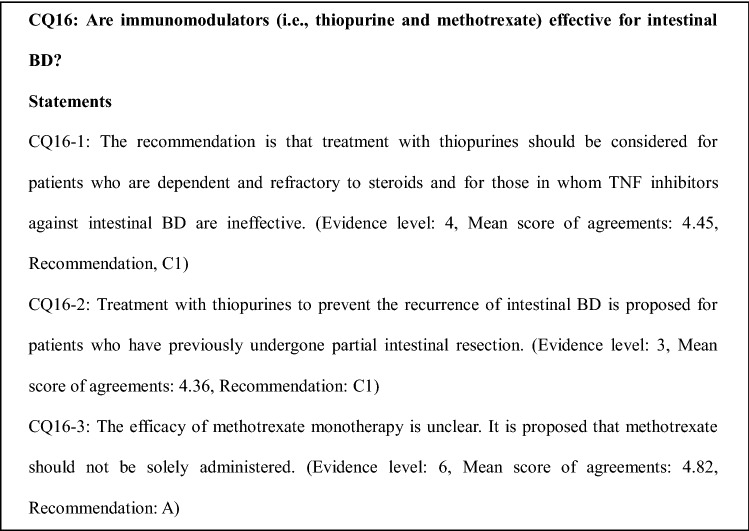


### Comments on CQ16

Treatment with thiopurines should be considered in patients who show steroid dependency and refractoriness and in those in whom TNF inhibitors against intestinal BD are ineffective [2]. However, no prospective study to date has demonstrated the efficacy of maintenance therapy with thiopurines. A single-center retrospective study that investigated 39 intestinal BD patients who continuously received maintenance therapy with thiopurines after drug- or surgery-induced clinical remission showed cumulative relapse rates of 5.8%, 28.7%, 43.7%, and 51.7% at 1, 2, 3, and 5 years, respectively. In addition, multivariate analysis showed that younger age (< 25 years) at diagnosis and lower hemoglobin level (< 11 g/dL) were independent predictive factors for relapse [49]. Another retrospective study that evaluated the influence of thiopurine-induced leukopenia on the long-term prognosis of 196 inflammatory bowel disease (IBD) patients, including 83 patients with intestinal BD who were treated with azathioprine (AZA)/6-mercaptopurine (MP) and achieved remission, showed a significantly higher cumulative relapse-free survival rate in the leukopenic group than in the non-leukopenic group for all types of IBDs, including ulcerative colitis (UC), Crohn’s disease (CD), and intestinal BD (log-rank test, *P* = 0.032, 0.047, and 0.002, respectively) [48]. One retrospective study that examined the clinical prognostic factors in 43 patients with intestinal BD showed a lower probability of undergoing repeat surgery in patients who underwent intestinal operation and subsequently took AZA than in those who did not (7% vs. 25% at 2 years and 25% vs. 47% at 5 years; *P* = 0.035) [36]. A single-center retrospective study that compared the clinical effects of thiopurines with those of 5-ASA in 77 intestinal BD patients who had undergone bowel resection and subsequently received 5-ASA (*n* = 50) or thiopurine (*n* = 27) therapy showed a lower postoperative recurrence rate in patients who received postoperative thiopurines than in those who did not (*P* = 0.050), with a hazard ratio of 0.636 (95% confidence interval 0.130–1.016, *P* = 0.053). However, the rates of repeat surgery were not significantly different between the 5-ASA and thiopurine groups [55].

Regarding the efficacy of methotrexate in patients with intestinal BD, a retrospective observation study that assessed the long-term (i.e., within 2 years) efficacy and safety of combination therapy with infliximab and methotrexate reported complete disappearance of ulcers and excellent tolerability in 9 of 10 patients with intestinal BD that was refractory to conventional therapies [41].
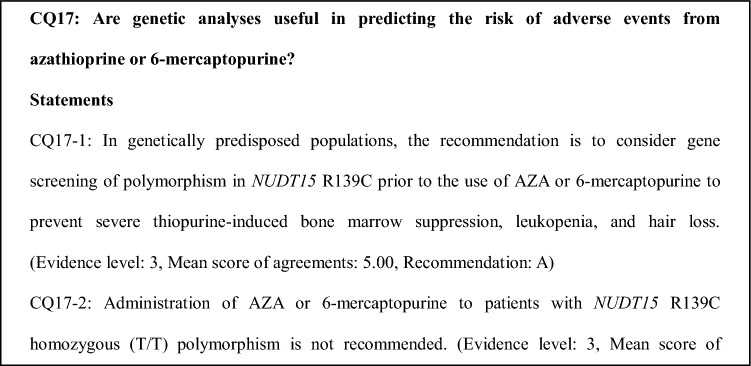


### Comments on CQ17

Adverse events associated with AZA or 6-MP treatment may be dose-dependent or independent. Dose-independent adverse events include fever, eruptions, arthralgia, muscle pains, pancreatitis, and digestive symptoms, whereas dose-dependent adverse events include liver dysfunction, delayed hair loss, nausea, and vomiting.

*NUDT* (nudix hydrolase)*15* R139C homozygous polymorphism is a genetic risk for thiopurine-induced severe leukopenia and hair loss, particularly in East Asian populations. This gene polymorphism is found in about 1% of East Asian populations [56–58]. However, it has not currently become clear that among patients of European ancestry with IBD, *NUDT15* R139C is associated with increased risk of thiopurine-induced leukopenia [59]. A multicenter retrospective pharmacogenetic study in Japan that investigated the association of *NUDT15* variants and haplotypes with adverse events in 2630 IBD patients (CD 1049, UC 1522, and BD 60) showed that *NUDT15* p. Arg139Cys was associated with leukopenia and hair loss, but was not closely associated with delayed leukopenia. Furthermore, thiopurine S-methyltransferase (TPMT) and other genes were not associated with leukopenia [60].

In the clinical setting, *NUDT15* R139C was not found to be useful in predicting adverse events other than thiopurine-induced severe leukopenia and hair loss. A retrospective cohort study that investigated the association of the risk for AZA-induced pancreatitis with the single nucleotide polymorphism mapped to the class II HLA gene, including the *HLA-DQA1* and *HLA-DRB1* haplotypes, in 373 IBD patients exposed to AZA showed that the class II HLA region was an important marker of the risk for AZA-induced pancreatitis [61]. However, there are no data at present to support this finding in Japanese IBD patients. Therefore, the class II HLA region is unlikely to be a useful predictive marker of AZA-induced pancreatitis in Japanese IBD patients.
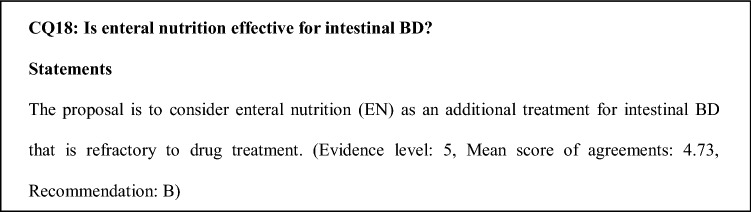


### Comments on CQ18

There is no evidence for the efficacy of EN therapy for intestinal BD. However, a case study in 12 intestinal BD patients reported that parenteral nutrition or EN therapy, including an elemental or low residual diet, promoted the resolution or disappearance of ulcers [62]. In addition, the second edition of the consensus statements for the diagnosis and management of intestinal BD proposed that EN could be considered, as EN with an elemental diet can be effective for induction therapy and is particularly indicated for patients with refractory disease, severe activity, and disability, such as those with stricture lesions. When EN is introduced, adherence and quality of life of the patients should be considered [2].
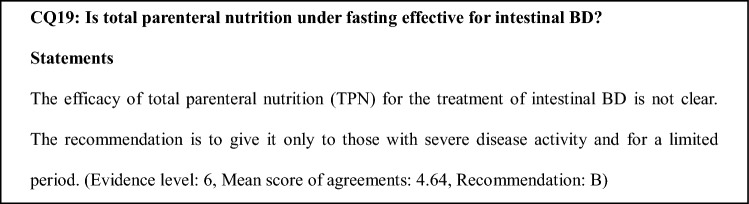


### Comments on CQ19

TPN is provided to patients with severe systemic symptoms, such as fever, or intestinal complications such as deep and giant ulcers, stenosis, fistula, bleeding, and imminent perforation. TPN is also provided to patients who cannot take drugs orally because of severe oral or upper gastrointestinal lesions. Considering the risk for catheter infection and venous thrombosis, TPN is typically applied for a limited period only [2]. The clinical situations associated with the highest risk for venous thromboembolism and catheter infection are dehydration and corticosteroid use [63].
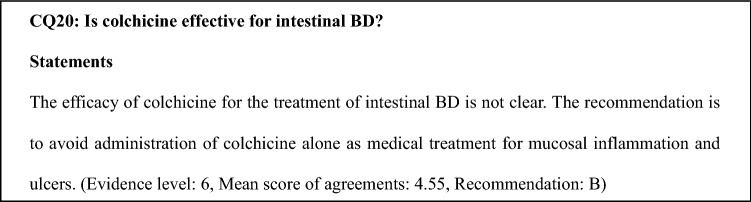


### Comments on CQ20

The efficacy of colchicine is experimentally limited and unclear, and it is because there is no evidence for its efficacy for the treatment of intestinal BD. The therapeutic use of colchicine is not limited to extra-intestinal manifestations.
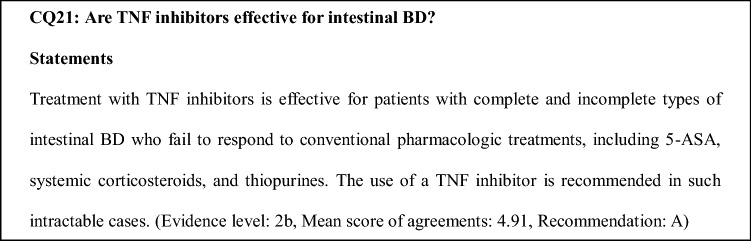


### Comments on CQ21

TNF inhibitors are effective for complete and incomplete types of intestinal BD [33, 34, 45, 64]. In intestinal BD patients with ileocecal ulcers measuring ≥ 1 cm in diameter, those who have severe gastrointestinal symptoms that affect activities of daily living, and those who are refractory to conventional medical treatments, the rates of complete disappearance of both gastrointestinal symptoms and ileocecal ulcers after initiating TNF inhibitors (infliximab or adalimumab) were 20.0%–54.5% at 24–30 weeks and 20.0%–60.0% at 52 weeks [33, 34].
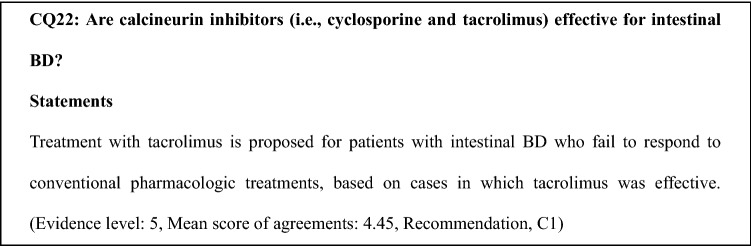


### Comments on CQ22

There has been only one case report to date on the effectiveness of a calcineurin inhibitor for intestinal BD. In this case of intestinal BD that failed to respond to 5-ASA, corticosteroids and cyclosporine, tacrolimus induced clinical remission and endoscopic healing [47]. However, it is important to note that treatment with a calcineurin inhibitor is contraindicated in patients with neurologic BD, as it can induce central neurologic disorders. Tacrolimus is not approved in Japan.

### Surgical treatment


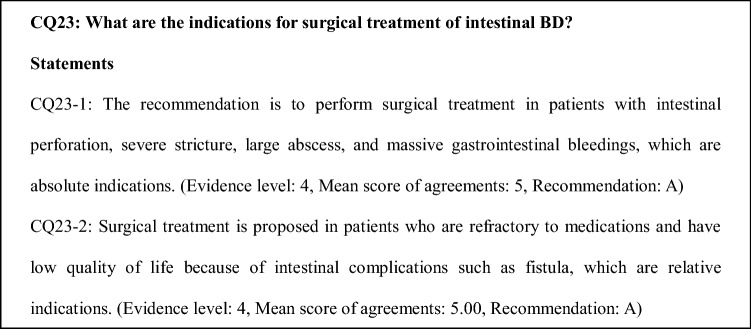


### Comments on CQ23

There is no evidence for the indication of surgical treatment for intestinal BD. Previous reports have demonstrated the use of surgical therapy in patients with intestinal perforations, lesions with severe stricture, large abscesses, and life-threatening massive gastrointestinal bleeding [25]. In addition, after counseling and communication with gastroenterologists and surgeons about the indications and postoperative prognosis, surgical therapy may be performed in patients who are refractory to medications and have low quality of life due to intestinal complications, such as fistula.

During or after surgery for acute appendicitis or intestinal perforation, a definite diagnosis of intestinal BD is often confirmed [25]. Extensive resection of the intestine to encompass multiple affected lesions has been shown to reduce the rate of postoperative recurrence [65]. However, a low rate of postoperative recurrence has also been found, even with a minimum length of resection [66]. Therefore, a minimum intestinal length for surgical resection should be considered [27, 66].
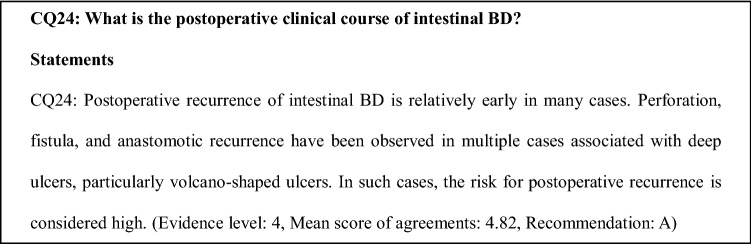


### Comments on CQ24

Postoperative recurrence within 2 years has been known to occur in many patients who have undergone surgical treatment [25, 66, 67]. In fact, the 2-year cumulative recurrence rate has been reported to be high, ranging from 30 to 75% [25, 28]. A report from Korea demonstrated the frequent need for repeat surgery for recurrent disease in long-term follow-up, with cumulative rates of 12.5% within 2 years and 22.2%–31% within 5 years [28].

Recurrence had been observed in many cases of patients with volcano-shaped deep ulcers [28], perforation or fistula [36, 68], abscesses or fistula formation from a ruptured anastomotic suture [27, 65], and postoperative corticosteroid use. Therefore, the number of cases that undergo multiple surgeries is not insignificant [25, 27, 66].
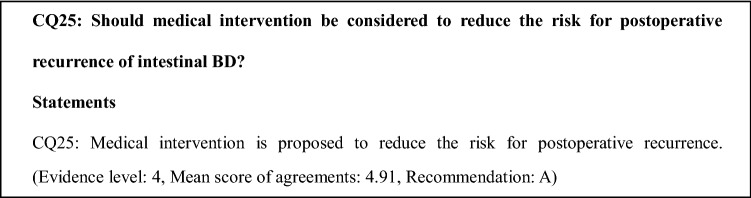


### Comments on CQ25

A treatment strategy to reduce the risk for postoperative recurrence and repeat surgery has not yet been established. Given the high rates of recurrence and repeat surgery in postoperative patients, medical management including enteral nutrition and drug therapy to maintain clinical remission is frequently required.

Volcano-shaped deep ulcers [2, 67], fistula [2], CRP value ≥ 4.4 mg/dL [67], and the presence of pathologically confirmed perforation [67] have been found to be predictive of high recurrence risk. Similarly, volcano-shaped deep ulcers [67], corticosteroid use [28, 67], postoperative complications [69], and difficulty in gaining weight [28] are predictive of high risk of repeat surgery. The reported cumulative rates for recurrence after surgical treatment were 29.2% at 2 years and 47.2% at 5 years, whereas those for repeat surgery were 12.5% at 2 years and 22.2% at 5 years [28]. In addition, there were no differences reported in cumulative probabilities for postoperative clinical recurrence at 10 years between CD and intestinal BD (66.5% vs. 79.1%, respectively, *p* = 0.724) [69]. Postoperative medical management should be considered in patients with the above-mentioned risk factors for recurrence and repeat surgery.

Notably, there is no evidence for the efficacy of drug therapy in postoperative medical management of intestinal BD. A retrospective study on the postoperative clinical outcomes of 77 patients with intestinal BD showed that compared with those who received 5-ASA, patients who received thiopurine had a lower postoperative recurrence rate (HR 0.636, 95% CI 0.130–1.016, *P* = 0.053) but similar rate of repeat surgery, readmission, and death [55]. Therefore, postoperative thiopurine treatment should be considered to prevent recurrence of intestinal BD in long-term follow-up.

### Special situations: Intestinal BD in childhood and trisomy 8


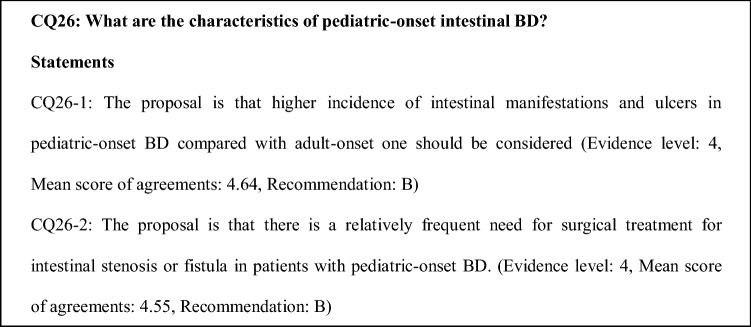



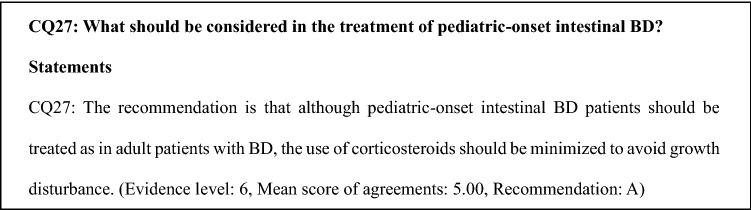


### Comments on CQ26 and 27

A 1997 nationwide retrospective survey that evaluated the incidence of BD showed that among 31 Japanese children with BD, the complete type was present in 3 cases, incomplete type in 24 cases, and suspected BD in 4 cases; furthermore 51.6% had gastrointestinal symptoms or lesions and 38.7% had imaging study-confirmed intestinal ulcers [70]. The fourth nationwide epidemiologic survey on BD in 1991 reported intestinal involvement in 15.5% of 3,316 patients with intestinal BD [71]. Therefore, the gastrointestinal manifestations of BD are thought to be more frequent in children than in adults.

A study that reviewed the clinical features of 22 Japanese pediatric-onset BD patients reported the frequency of oral ulcers (100%), skin manifestations (72.7%), genital ulcers (72.7%), and ocular involvement (22.7%). As for adult-onset intestinal BD, patients with pediatric-onset intestinal BD had ocular involvement less frequently than in other types of BD. Furthermore, 54.5% of pediatric-onset intestinal BD reportedly required surgical treatment for intestinal stenosis and perforation [72]. Case series and retrospective studies on the clinical spectrum of BD in Western countries reported more common gastrointestinal symptoms of abdominal pain and diarrhea in children than in adults. However, these reports did not reflect the incidence of intestinal BD, since endoscopic findings were not confirmed [73–75].

There is no evidence to date for the efficacy of medical treatment for pediatric-onset intestinal BD. Some case series have reported the efficacy of 5-ASA, corticosteroids, immunomodulators, enteral nutrition, colchicine, TNF inhibitors, and thalidomide in children with intestinal BD [76, 77]. In addition, some case reports have described the efficacy of bone marrow transplantation and surgery in children with BD [78]. Excessive use of corticosteroids cannot be justified in children with intestinal BD since growth may be affected.
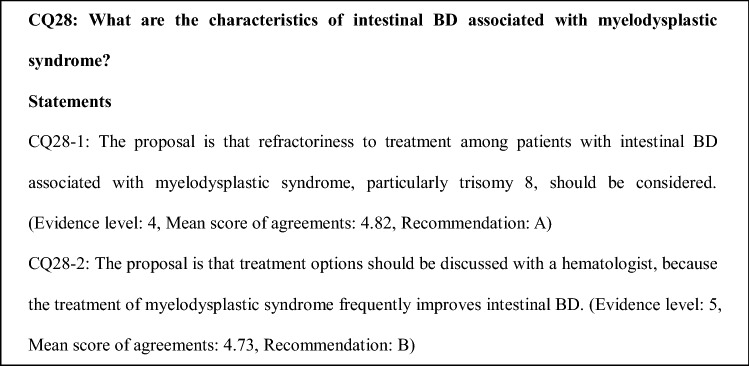


### Comments on CQ28

BD associated with MDS tends to have gastrointestinal involvement [79, 80]. An analysis of 70 patients with Warkany syndrome demonstrated the presence of chromosomal trisomy 8 allele in about 10% of patients with primary MDS [81]. Moreover, 54%–86% of intestinal BD patients with MDS were reported to harbor the trisomy 8 allele [7, 8, 80]. However, the association of intestinal BD with trisomy 8 remains unclear, given the lack of detailed reports on the incidence of endoscopically-confirmed intestinal ulcers in patients with MDS or intestinal BD with MDS. Therefore, further accumulation of data on the incidence of intestinal ulcers is required.

Some cases present with intestinal BD followed by MDS, while others present with MDS followed by intestinal BD [8]. When pancytopenia is discovered in patients with intestinal BD, the contributory causes include drug-induced MDS and episodic occurrence of MDS.

Myeloablative allogeneic hematopoietic stem cell transplantation (HSCT) has been reported to be successful in several patients with refractory intestinal BD with MDS, resulting in complete resolution of both BD and MDS. A systematic review of HSCT in 20 patients with BD showed that 6 patients underwent HSCT because of accompanying MDS; 4 of these 6 patients had refractory intestinal BD and achieved complete remission [82]. Intestinal BD associated with MDS and trisomy 8 is generally refractory to conventional medical therapies. TNF inhibitors for intestinal lesion and azacytidine for MDS have been reported to be effective for both intestinal and hematological conditions in some cases [83, 84].

## Abbreviations

5-ASA: 5-Aminosalicylic acid
6-MP: 6-Mercaptopurine
AZA: Azathioprine
BD: Behçet’s disease
CD: Crohn’s disease
CQ: Clinical question
CRP: C-reactive protein
CT: Computed tomography
DAIBD: Disease activity index for intestinal Behçet’s disease
EN: Enteral nutrition
HLA: Human leukocyte antigen
HSCT: Hematopoietic stem cell transplantation
IBD: Inflammatory bowel disease
MINDS: Medical information network distribution service
MDS: Myelodysplastic syndrome
MRI: Magnetic resonance imaging
MTX: Methotrexate
NSAID: Non-steroidal inflammatory drug
NUDT 15: Nudix hydrolase 15
SASP: Salazosulfapyridine
TNF: Tumor necrosis factor
TPMT: Thiopurine S-methyltransferase
TPN: Total parenteral nutrition
